# Clostridium perfringens enterotoxin carboxy-terminal fragment is a novel tumor-homing peptide for human ovarian cancer

**DOI:** 10.1186/1471-2407-10-349

**Published:** 2010-07-02

**Authors:** Emiliano Cocco, Francesca Casagrande, Stefania Bellone, Christine E Richter, Marta Bellone, Paola Todeschini, Jennie C Holmberg, Han Hsuan Fu, Michele K Montagna, Gil Mor, Peter E Schwartz, Dan Arin-Silasi, Masoud Azoudi, Thomas J Rutherford, Maysa Abu-Khalaf, Sergio Pecorelli, Alessandro D Santin

**Affiliations:** 1 Department of Obstetrics, Gynecology & Reproductive Sciences, Yale University School of Medicine, New Haven, CT, USA; 2 Internal Medicine & Oncology, Yale University School of Medicine, New Haven, CT, USA; 3 Division of Gynecologic Oncology, University of Brescia, Brescia, Italy

## Abstract

**Background:**

Development of innovative, effective therapies against recurrent/chemotherapy-resistant ovarian cancer remains a high priority. Using high-throughput technologies to analyze genetic fingerprints of ovarian cancer, we have discovered extremely high expression of the genes encoding the proteins claudin-3 and claudin-4.

**Methods:**

Because claudin-3 and -4 are the epithelial receptors for *Clostridium perfringens enterotoxin *(CPE), and are sufficient to mediate CPE binding, in this study we evaluated the *in vitro *and *in vivo *bioactivity of the carboxy-terminal fragment of CPE (i.e., CPE_290-319 _binding peptide) as a carrier for tumor imaging agents and intracellular delivery of therapeutic drugs. Claudin-3 and -4 expression was examined with rt-PCR and flow cytometry in multiple primary ovarian carcinoma cell lines. Cell binding assays were used to assess the accuracy and specificity of the CPE peptide *in vitro *against primary chemotherapy-resistant ovarian carcinoma cell lines. Confocal microscopy and biodistribution assays were performed to evaluate the localization and uptake of the FITC-conjugated CPE peptide in established tumor tissue.

**Results:**

Using a FITC-conjugated CPE peptide we show specific *in vitro *and *in vivo *binding to multiple primary chemotherapy resistant ovarian cancer cell lines. Bio-distribution studies in SCID mice harboring clinically relevant animal models of chemotherapy resistant ovarian carcinoma showed higher uptake of the peptide in tumor cells than in normal organs. Imunofluorescence was detectable within discrete accumulations (i.e., tumor spheroids) or even single chemotherapy resistant ovarian cancer cells floating in the ascites of xenografted animals while a time-dependent internalization of the FITC-conjugated CPE peptide was consistently noted in chemotherapy-resistant ovarian tumor cells by confocal microscopy.

**Conclusions:**

Based on the high levels of claudin-3 and -4 expression in chemotherapy-resistant ovarian cancer and other highly aggressive human epithelial tumors including breast, prostate and pancreatic cancers, CPE peptide holds promise as a lead peptide for the development of new diagnostic tracers or alternative anticancer agents.

## Background

Ovarian carcinoma remains the most lethal of gynecologic malignancies [1]. Although many patients with ovarian cancer fully respond to the standard combination of surgery and chemotherapy, nearly 90% later develop recurrent chemotherapy-resistant cancer and inevitably succumb to their disease [2] Thus, development of novel, effective therapies against recurrent/chemotherapy-resistant ovarian cancer remains a high priority. As an early step toward our long-term goal to develop an innovative and highly effective therapeutic strategy for patients with chemotherapy-resistant ovarian cancer, we sought molecular targets to distinguish healthy cells from ovarian cancer cells. Using oligonucleotide microarrays to analyze global gene expression profiles of several highly purified primary ovarian serous papillary tumor (OSPC) cell lines, we and others have consistently identified the genes encoding tight junction (TJ) proteins claudin-3 and -4 as two of the most highly upregulated genes in primary ovarian carcinoma [3-13].

Claudin-3 and -4 are the epithelial receptors for *Clostridium perfringens enterotoxin *(CPE), a potent cytolytic toxin [14-16]. CPE triggers lysis of mammalian epithelial cells through interactions with claudin-3 and -4 receptors while cells that do not express CPE receptors (i.e., mesothelial cells and most healthy human tissues) are protected from the lethal effects of CPE because they fail to bind the toxin. Our previous studies suggest that chemotherapy-resistant ovarian tumors express extremely high levels of claudin-3 and -4 and that these tumors are highly sensitive to CPE *in vitro *[17]. Unfortunately, the systemic (i.v.) administration of CPE in animals is toxic, limiting this approach to regional/local therapy [16]. Importantly, functional domain mapping of the full length 319-amino acid CPE protein demonstrated that the carboxy-terminal fragment (C-CPE_290-319 _peptide) is sufficient for high-affinity binding to target cell receptors and small complex formation, although this fragment is not toxic because it is incapable of initiating large complex formation and subsequent cytolysis [15]. Because peptides are promising molecules to deliver radionuclides or chemotherapeutic drugs into tumors, we hypothesized that using the C- CPE_290-319 _peptide to target ovarian cancer cells based on their high levels of claudin-3 and -4 may represents a novel, potentially highly effective diagnostic and therapeutic approach to identify and treat chemotherapy-resistant ovarian cancer.

In this study we have carefully assessed the accuracy and specificity of the CPE peptide *in vitro *in multiple cell binding assays against primary chemotherapy-resistant ovarian carcinoma cell lines. Confocal microscopy experiments showed tumor internalization of the CPE peptide in a time-dependent manner. Biodistribution experiments in highly relevant clinical models of chemotherapy-resistant freshly explanted human ovarian cancer in SCID mouse xenografts were done demonstrating high uptake of the FITC-conjugated CPE peptide in established tumor tissue as well as tumor spheroids floating in the ascitic fluid, recommending this peptide as a promising lead structure for improved targeting of chemotherapy-resistant ovarian cancer.

## Methods

### Peptide

The *Clostridium Perfringens *carboxy-terminal fragment peptide (CPE_290-319 _peptide) was synthesized by InVitrogen Corporation (Invitrogen, Grand Island, N.Y.), with the sequence SLD AGQ YVL VMK ANS SYS GNY PYS ILF QKF and a purity level >95%. The CPE peptide and its FITC-conjugated form were purified by high performance liquid chromatography and their sequence and structure were confirmed by mass spectrometry.

### Primary Chemotherapy-Resistant Ovarian Carcinoma Cell Lines

Primary ovarian carcinoma cell lines used in this study were established from samples obtained either at the time of primary surgery or collected at the time of tumor recurrence from patients harboring advanced stage ovarian carcinoma. All primary cell lines were evaluated for claudin-3 and claudin-4 expression by real time-PCR and flow cytometry. Five primary high grade serous papillary carcinomas (OSPC) and two clear cell ovarian cancers (CC) were available for this study. The characteristics of the primary specimens used in our experiments are depicted in Table 1. With the exception of CC-ARK-2 all other primary tumor specimens, including OSPC-ARK-1, a fresh ovarian serous papillary carcinoma used to establish ovarian xenografts in SCID mice, were confirmed to be highly resistant to multiple chemotherapeutic agents when measured as percentage cell inhibition (PCI) by *in vitro *Extreme Drug Resistance (EDR) assay (Oncotech Inc. Irvine, CA) [18]. Other control cell lines evaluated in the CPE peptide binding assays included Vero cells (green monkey kidney cells), human fibroblasts, primary squamous and adenocarcinoma cervical cancer cell lines and Epstein-Barr transformed B lymphocytes (LCL). All fresh specimens were cultured in RPMI 1640 medium (Invitrogen) containing 10% fetal bovine serum (FBS; Gemini Bio-products, Calabasas, CA), 200 u/ml penicillin, and 200 μg/ml streptomycin, as previously described [17]. All samples were obtained with appropriate consent according to IRB guidelines. The epithelial nature and the purity of epithelial tumor cultures were verified by immunohistochemical staining and flow cytometric analysis with antibodies against cytokeratin as previously described [17].

**Table 1:** Claudin-3 and -4 mRNA and protein expression in primary ovarian cancer cell lines

Cell line	Patient characteristics		Flow cytometry		rtPCR	
	
	Histology	Stage	Claudin-3/4	Claudin-3/4	Claudin-3	Claudin-4
	
			MFI* + SD	Cells (%)	mRNA copy #	mRNA copy #
NOVA**			11.2 ± 3.4	5	1	1

VERO cells			86.8 ± 10.5	100	-	-

OSPC-ARK-1	OSPC***	IV	118.8 ± 12.5	100	6654	2372

OSPC-ARK-2	OSPC	IIIC	58.7 ± 6.1	100	4124	962

OSPC-ARK-3	OSPC	IIIA	89.8 ± 8.7	100	31129	330

OSPC-ARK-4	OSPC	IV	45.9 ± 16.2	100	37122	140

OSPC-ARK-5	OSPC	IV	102.7 ± 11.3	100	9108	6418

CC-ARK-1	CC****	IIIC	108.3 ± 8.7	100	7559	325

CC-ARK-2	CC	IC	105.7 ± 10.2	100	10141	2055

*MFI: Mean Fluorescence Intensity

**NOVA: Normal Ovarian control cells

***OSPC: Ovarian Serous Papillary Carcinoma

****CC: Clear Cell ovarian carcinoma

### RNA Extraction and Quantitative Real-time PCR

RNA isolation from primary cell lines was performed using TRIzol Reagent (Invitrogen) according to the manufacturer's instructions. q-PCR was performed with an ABI Prism 7000 Sequence Analyzer using the manufacturer's recommended protocol (Applied Biosystems, Foster City, CA) to evaluate expression of claudin-3 and claudin-4 in all the samples. Each reaction was run in triplicate. Briefly, five μg of total RNA from each sample was reverse transcribed using SuperScript III first-strand cDNA synthesis (Invitrogen, Carlsbad, CA). Five μl of reverse transcribed RNA samples (from 500 μl of total volume) were amplified by using the TaqMan Universal PCR Master Mix (Applied Biosystems) to produce PCR products specific for claudin-3 and claudin-4. The primers for claudin-3 and claudin-4 were obtained from Applied Biosystems as Assay-on-Demand products. Assays ID were Hs00265816_s1 (claudin-3) and Hs00433616_s1 (claudin-4). The comparative threshold cycle (*C*_T_) method (PE Applied Biosystems) was used to determine gene expression in each sample relative to the value observed in the nonmalignant ovarian epithelial cells, using GAPDH (Assay-on-Demand Hs99999905_m1) RNA as internal controls.

### Flow Cytometry

We used FACS analysis to evaluate binding of the FITC-labeled C-CPE peptide to the surface of primary chemotherapy-resistant ovarian carcinoma cell lines overexpressing claudin-3 and -4 receptors. As positive control, we used a single chain fragment variable (scFv) specific against claudin-3 recently developed and characterized in our laboratory [19]. Negative controls included normal cells (i.e., peripheral blood lymphocytes (PBL), Epstein-Barr transformed B lymphocytes (LCL) and human fibroblasts) and tumor cell with low and/or negligible claudin-3 and -4 receptor expression (i.e., primary squamous and adenocarcinoma cervical cancer cell lines). Briefly, approximately 2.5 × 10^5 ^cells were resuspended with 50 μL PBS 1% bovine serum albumin (BSA) that contained 4 μg of FITC-labeled C-CPE peptide or primary scFv antibody (positive control) and cells were then incubated for 1 hour at room temperature. For scFv antibody staining, after further washing, cells were resuspended with 100 μL PBS 1% BSA solution that contain 0.7 μg of an anti-myc tag antibody 9E10 (Roche Diagnostics) for 1 hour at 4°C, followed by washing and incubation with 2 μg of fluorescein isothiocyanate (FITC)-conjugated goat antimouse immunoglobulin G (Jackson Immuno Research, West Grove, PA) for 30 minutes at 4°C. Cells stained with FITC-labeled C-CPE peptide or scFv and unstained control cells were then washed with PBS, resuspended in 500 μL PBS, and immediately analyzed by FACScalibur (BD Biosciences, San Diego, CA).

### Fluorescent Microscopy

The surface binding localization of FITC-conjugated-C-CPE_290-319 _peptide on chemotherapy resistant ovarian tumors was further evaluated by fluorescence microscopy. Briefly, unfixed ovarian cancer cells or control cells in suspension or adherent to plastic were incubated with different concentrations of FITC-conjugated-C-CPE_290-319 _peptide for 30 to 60 minutes at 37°C and binding to cell surface expressed claudin-3 and claudin-4 was analyzed by fluorescent microscopy using a Nikon fluorescence microscope Eclipse E400. Images were captured digitally and analyzed using Metamorph 3.6a version software.

### Internalization experiments and Confocal laser scanning microscopy

We used confocal microscopy to evaluate whether the FITC-conjugated-C-CPE _290-319 _peptide was able to internalize after binding to claudin-3 and claudin-4 receptors on the surface of chemotherapy-resistant ovarian cancer cells. Briefly, 30,000 ovarian cancer cells were seeded in 3 cm petri dishes. After 24 hours of cultivation, the medium was replaced by fresh medium and FITC-conjugated CPE _290-319 _peptide (10 μg/ml) was added to the cells. The FITC-labeled peptide was incubated with the tumor cells for the indicated times (i.e., from 30 to 60 minutes at 37°C). The medium was then removed and the cells were washed thrice with 1 mL PBS before being analyzed by confocal imaging on an inverted microscope (Leica DM IRBE) with a confocal laser scanning unit (Leica SP2 MP). Some of the experiments were also carried out using the unlabeled CPE _290-319 _peptide as competitor for the binding of FITC-conjugated CPE_290-319 _peptide while samples without FITC-conjugated CPE _290-319 _peptide were analyzed to determine autofluorescence of tumor cells. Processed serial sections were constructed into three-dimensional images using VoxelView (Vital Images Ltd., Fairfield, IA) on a Silicon Graphics Indy workstation (Mountain View, CA).

### Tumor targeting in SCID Mouse Tumor Xenografts

C.B-17/SCID female mice 5-7 weeks old were obtained from Harlan Sprague-Dawley (Indianapolis, IN) and housed in a pathogen-free environment at Yale University. They were given commercial basal diet and water ad libitum. The experimental protocol for the use of the animals for these studies was approved by the Institutional Animal Care and Use Committee. Animals were used to generate ovarian tumor xenografts as previously described [17]. Briefly, OVA-ARK-1 cancer cell line was injected intraperitoneally (i.p.) at a dose of 5 × 10^6 ^into C.B-17/SCID mice in groups of three to five. Six weeks after i.p. tumor injection when carcinomatosis nodules had grown to 0.5 to 0.8 cm, 100 μg FITC-CPE peptide was injected into the tail vein and allowed to circulate for 12, 24 or 48 hours. In some experiments mice were perfused at the time of sacrifice with PBS through the left ventricle to remove blood and unbound peptide. Tumors and control organs were excised after the injection of the fluorescent peptide and examined for fluorescence using a whole-body optical imaging system. Selective excitation of FITC was produced through a D455/70 nm band-pass excitation filter (HB Optical). Emitted fluorescence was collected through a long-pass filter (520 nm; HB Optical) on a 3.3 MP digital camera (DC290; Kodak).

### Statistics

Statistical comparisons between groups were done by the unpaired Student's t test using SPSS version 15 (SPSS, Chicago, IL). P ≤ 0.05 was considered statistically significant.

## Results

### Claudin-3 and Claudin-4 Transcript Levels in Chemotherapy-Resistant Ovarian Tumors

We used q-RT-PCR assays to get highly sensitive measurements of claudin-3 and claudin-4 CPE receptor expression in normal tissue cells and primary human tumors derived from patients harboring chemotherapy-resistant ovarian carcinomas. Both claudin-3 and/or claudin-4 genes were found highly expressed in all primary ovarian cancer studied including five high grade serous papillary tumors and two high grade clear cell carcinomas when compared to normal ovarian epithelial cells (NOVA) as well as other normal cells or other gynecologic tumors (i.e., mean claudin-3 expression = 15,119 folds and mean claudin-4 expression = 1,800 folds, respectively, when compared to NOVA, Table 1, p < 0.05, and data not shown). These results are consistent with previous reports from us as well as others, showing high expression of claudin-3 and claudin-4 in ovarian carcinomas by gene expression profiling [3-13], and confirm high expression of the CPE receptor transcripts in chemotherapy-resistant ovarian tumors.

### Flow Cytometry experiments

**Figure 1:**
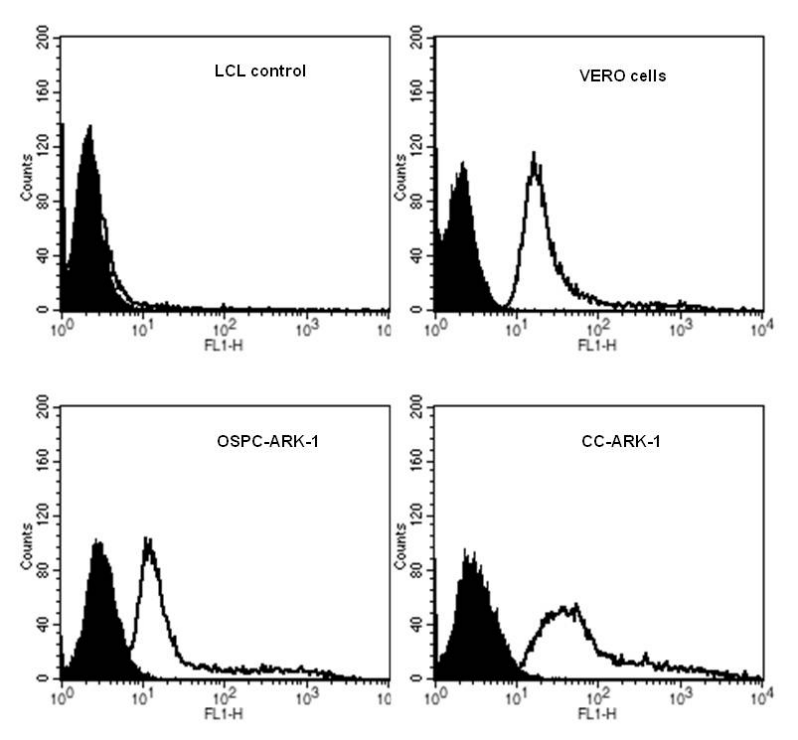
**Representative flow cytometry histograms showing claudin-3/4 expression in primary chemotherapy resistant ovarian carcinoma cell lines after labeling with FITC-conjugated CPE peptide**. LCL (negative control), Vero cells (positive control), OSPC-ARK-1 (primary OSPC cell line) and CC-ARK-1 (primary clear cell ovarian carcinoma cell line). Both ovarian chemotherapy-resistant tumors demonstrated high level of claudin-3 and claudin-4 expression. Isotype (solid black peak); FITC-conjugated CPE peptide; (black line).

On the basis of the high expression of claudin-3 and/or claudin-4 detected by RT-PCR in primary ovarian cancer cell lines we predicted that the FITC-conjugated CPE peptide may bind to ovarian tumors *in vitro*. We conducted a set of experiments to demonstrate this directly on fresh human ovarian carcinoma cells in a clinically relevant setting of ovarian cancer disease for which current salvage therapy is ineffective (i.e., chemotherapy-resistant ovarian cancer). To accomplish this goal, we evaluated the ability of the FITC-conjugated CPE peptide to label the freshly established primary ovarian tumor cultures in flow cytometry experiments; in parallel, we also evaluated VERO cells (positive controls, [20]), and healthy ovarian cells, fibroblasts, peripheral blood lymphocytes (PBL), lymphoblastoid cell lines (LCL), and healthy human keratinocytes, which do not express detectable levels of claudin-3 or -4 (negative controls). As representatively demonstrated in Figure 1 for two human primary chemotherapy resistant ovarian cancer cell lines (i.e., OSPC-ARK-1 and CC-ARK-1), FITC-conjugated CPE peptide was found highly reactive against all primary ovarian tumor cell lines, with positivity in 100% of tumor cells and a mean fluorescence intensity (MFI) value similar or superior to that found on VERO cells (Figure 1, Table 1). In contrast, negligible or no binding was detected by flow cytometry against LCL derived from the same patient from which OSPC-ARK-1 primary chemotherapy resistant tumor cell line was established (Figure 1) or normal ovarian cells expressing low levels of claudin-3 and/or -4 (i.e., NOVA, Table 1). While the data are not shown, a high level of expression for claudin-3 was further confirmed in OSPC-ARK-1 and CC-ARK-1 by flow cytometry using a human single-chain antibody fragment against claudin-3 (scFv, i.e., positive control) recently developed and characterized by our research group [19].

### FITC-labeled CPE peptide binds and compartmentalizes on the surface of chemotherapy-resistant ovarian tumor cells in vitro

**Figure 2:**
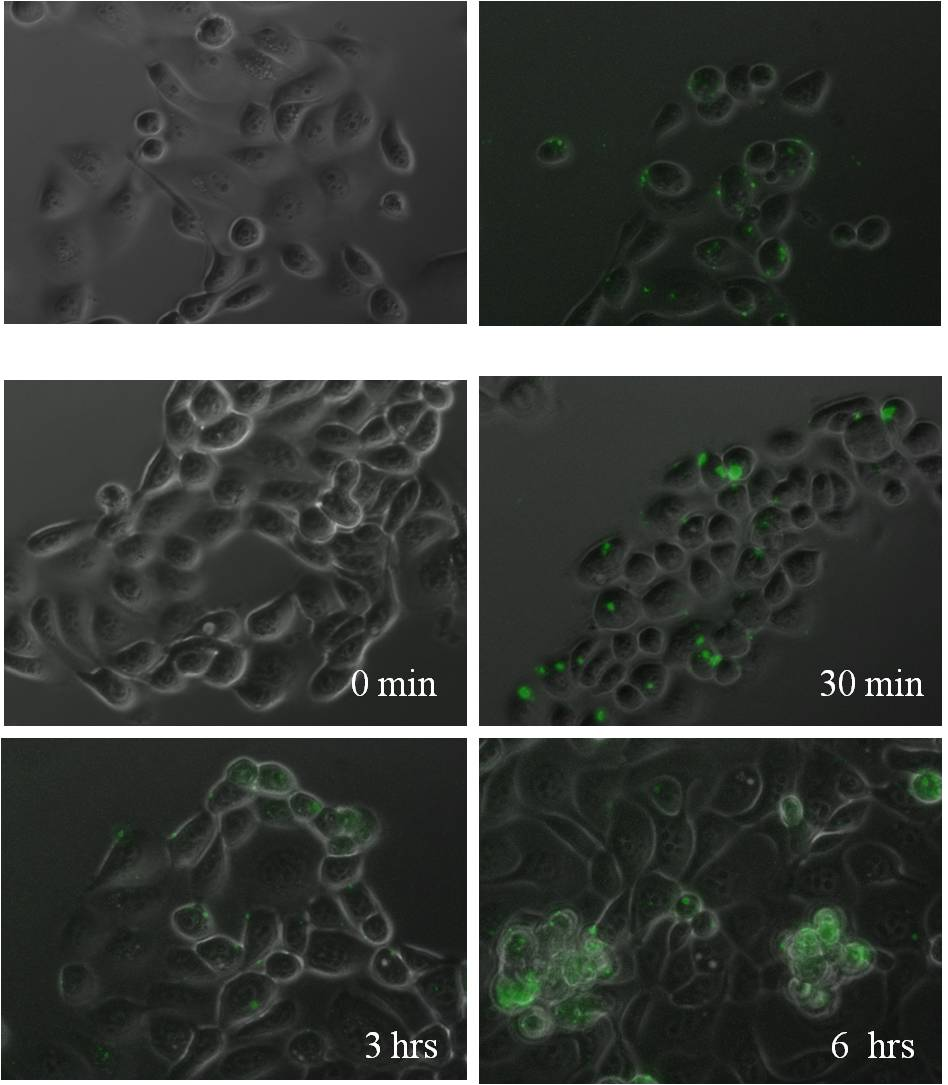
**Representative fluorescence microscopy pictures of claudin-3/4 expression in OSPC-ARK-1 and CC-ARK-1 primary chemotherapy resistant ovarian carcinoma cells**. **Upper panel**: Left: OSPC-ARK-1 control cells before exposure to FITC-conjugated CPE peptide. Note the lack of intrinsic fluorescence in the primary ovarian carcinoma. Right: OSPC-ARK-1 cells 30 minutes after the exposure to 10 μg/ml of FITC-conjugated CPE peptide. Note the presence of a punctuate fluorescence pattern on tumor cells suggesting that FITC-conjugated CPE peptide is compartmentalized after entry into tumor cells. **Lower panels**: CC-ARK-1 control cells before exposure to FITC-conjugated CPE peptide and 30 minutes, 3 and 6 hours, respectively, after the exposure to 10 μg/ml of FITC-conjugated CPE peptide. Note the presence of a punctuate fluorescence pattern on tumor cells suggesting that FITC-conjugated CPE peptide is compartmentalized after entry into tumor cells.

We used fluorescence microscopy to determine whether CPE peptide binds to primary chemotherapy resistant tumor cell lines *in vitro*. As representatively shown for OSPC-ARK-1, a high grade serous papillary ovarian carcinoma (Figure 2 upper panel), fluorescein imaging revealed that CPE peptide bound to all highly chemotherapy-resistant tumor cell lines overexpressing claudin-3 and/or -4 while lack of intrinsic fluorescence was noted in the primary ovarian carcinoma tested (Figure 2 upper panel). In contrast, in agreement with the flow cytometry results presented in Figure 1, CPE peptide did not bind to normal B cells (LCL) or tumor cells not expressing claudin-3 and/or claudin-4 (not shown). Importantly, we found the fluorescence intensity to be time- and dose dependent. As representatively shown for CC-ARK-1(Figure 2 Lower panels), a high grade clear cell ovarian carcinoma, binding of FITC-conjugated-peptide to the cellular surface of ovarian tumor cells was consistently detected at 30 minutes after exposure and continued to increase at 3 hrs and 6 hours, suggesting that the peptide is internalized after surface binding. Of interest, as shown in Figure 2, FITC-conjugated-CPE peptide-stained cells revealed a specific staining pattern, with claudin-3 and claudin-4 found distributed as focal and localized spots on the tumor cell surface. This pattern strongly suggests that FITC-conjugated CPE peptide may be compartmentalized after entry into tumor cells.

### FITC-labeled CPE peptide specifically binds and internalizes in chemotherapy-resistant ovarian tumor cells *in vitro*

**Figure 3:**
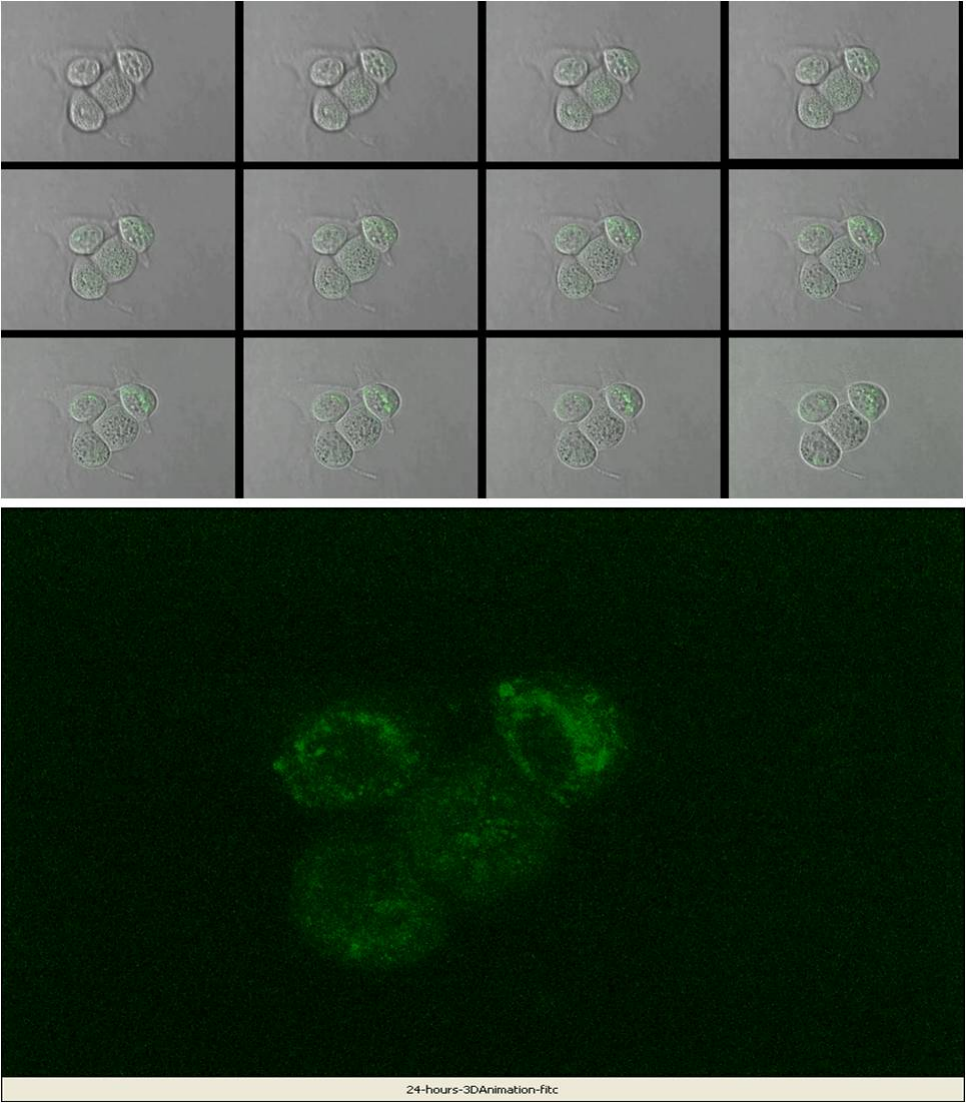
**Confocal microscopy analysis of intracellular distribution of FITC-conjugated CPE peptide in OSPC-ARK-1**. Tumor cells were obtained, exposed to FITC-conjugated CPE peptide and then evaluated by confocal microscopy (upper panel) and three-dimensional (3D) image reconstruction (lower panel). Images were reconstructed from confocal micrographs taken every 4 μm from the bottom of the culture slide up, and all views are perpendicular to the plane of the culture slide. Green fluorescence represents tumor staining with FITC-conjugated CPE peptide. The figures depict images reconstructed from multiple optical sections (total 40 μm) through the entirety of the cells. The upper panel figure shows corresponding images of the same cells at different levels of optical sectioning, and serves to highlight the "appearance" of FITC-conjugated CPE peptide material within the cytoplasm. In the lower panel representative 3D reconstruction images are shown, demonstrating dense aggregates of FITC-conjugated CPE peptide after 24 hours of peptide exposure in the cytoplasm of OSPC-ARK-1 cells.

We next evaluated by confocal microscopy whether the FITC-conjugated CPE peptide is able to internalize after binding to claudin-3 and claudin-4 receptors on the surface of chemotherapy-resistant ovarian cancer cells. Confocal images showed that the FITC-CPE peptide was bound to OSPC-ARK-1 cell membranes 5 to 10 min after incubation and was gradually internalized into the cytoplasm after 30 min. As shown in Figure 3, an intensive fluorescence signal in the form of irregular fluorescent clusters at the periphery and in the cytoplasm of the tumor cells was consistently noted after 6 h incubation while no staining was detected in LCL or other control cells (not shown). That FITC-conjugated CPE peptide was internalized, and not merely associated with the outer membrane surface, is revealed by the ability to detect the peptide only in optical sections through the tumor cell cytoplasm, but not in holistic reconstructions of whole cells where the tumor cell cytoplasm and plasma membrane completely surrounds the peptide in three-dimensional space (Figure 3). Indeed, with the use of confocal microscopy and 3D image reconstruction we were able to unequivocally demonstrate the internalization of the FITC-conjugated CPE peptide in primary cultured of OSPC-ARK-1 cells (Figure 3, lower panel) as well as the multiple other primary ovarian cancer cell lines available to this study (data not shown). Being able to view cell-associated FITC-conjugated CPE peptide in three-dimensional space uniquely allowed for resolution of completely internalized peptide from that which was only surface-bound or partially ingested. In contrast, after incubation with excess of unlabeled CPE peptide as competitor, reduced FITC fluorescence was detected on the membrane and/or cytoplasm while no positive staining was detected in OSPC-ARK-1 cells in the absence of the fluorescent peptide (not shown).

### Determination of FITC-conjugated-CPE peptide binding in biodistribution studies *in vivo*

**Figure 4:**
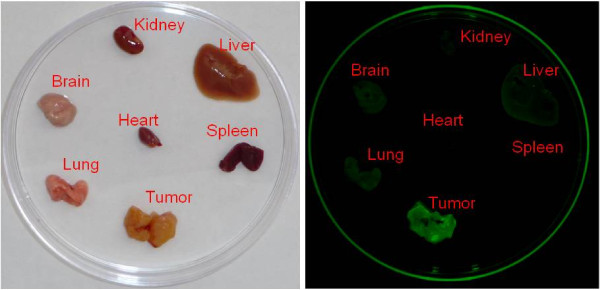
**FITC-conjugated CPE peptide distribution in OSPC-ARK-1 derived tumor xenografts in vivo**. Briefly, SCID ovarian tumor-bearing mice were injected with 100 μg FITC-conjugated CPE peptide i.v. 6 weeks after OSPC-ARK-1 transplantation. Experimental and control mice were anesthetized and sacrificed 12, 24 and 48 hrs later and the abdominal wall was opened and tumor and normal organs analyzed by confocal microscopy. The representative distribution of FITC-conjugated CPE peptide at 12 hrs after injection in dissected tumor and normal organs including kidney, liver, spleen, lungs, brain and heart is shown. Note the strong fluorescence accumulation in the tumor when compared to the control organs.

**Figure 5:**
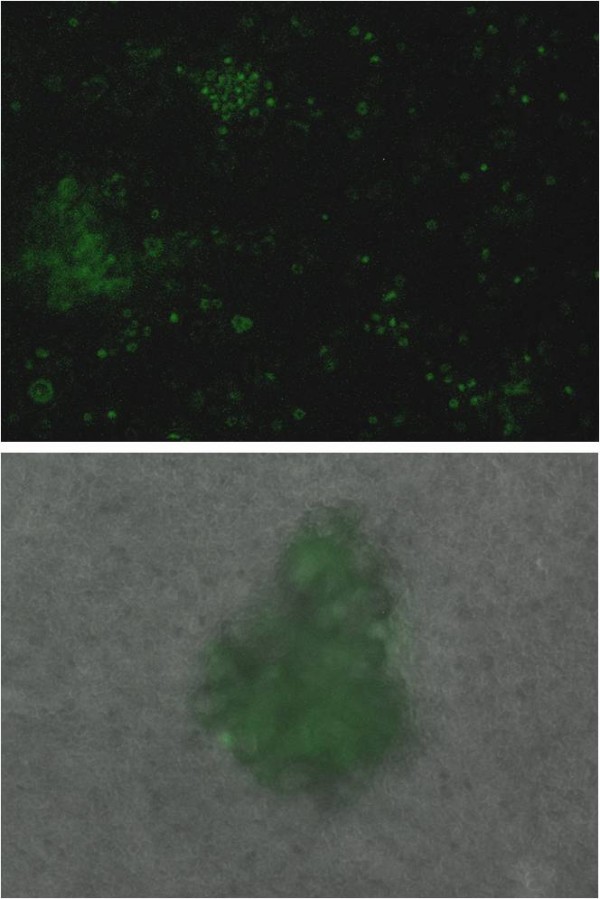
**FITC-conjugated CPE peptide localization in OSPC-ARK-1 derived tumor xenografts in vivo**: **Upper panel**: OSPC-ARK-1-derived tumor cells collected from the mouse ascites showed fluorescing tumor cells when analyzed as single cell suspension or as spherocytes (**Lower panel**) by fluorescence microscopy. Please note the fluorescent negative background constituted by the host contaminant inflammatory cells (i.e., tumor associated lymphocytes, macrophages and DC).

To determine the distribution of the FITC-conjugated CPE peptide in tumor bearing animals and whether FITC-conjugated CPE peptide is able to localize to tumor cells *in vivo*, SCID mice harboring OSPC-ARK-1-derived tumor xenografts 6-weeks after tumor implantation were injected via the tail vein (i.v.) with FITC-conjugated CPE peptide (100 μg). The peptide was then allowed to circulate in the blood stream for various time periods ranging from 12 to 48 hrs before tumors, ascites and controls organs were excised and processed from experimental and control animals. Mean fluorescence intensity was calculated over the ovarian tumors as well as multiple explanted organs/tissues including lungs, liver, spleen, brain, heart and kidney at 12 hrs, 24 hrs and 48 hrs after subtraction of background. Strong and specific fluorescence was detected in OSPC-ARK-1-derived tumor xenografts after12 hrs (mean MFI ± SE = 1178 ± 406), 24 hrs (mean MFI ± SE = 2592 ± 1303), and 48 hrs (mean MFI ± SE = 1808 ± 677) after i.v. injection with FITC-conjugated CPE peptide (Figure 4 and Table 2), while little labeling of tumor cells was observed after injecting equimolar concentrations of fluorescein, or fluorescein and unlabeled CPE peptide separately into mice with size-matched ovarian tumors or tumors not overexpressing claudin-3 and clauidn-4 receptors (data not shown). Table 2 depicts the time course of the tumor-to-background ratios at different time points after the i.v. injection of the FITC-conjugated CPE peptide. Remarkably, as shown in Figure 5, we found even discrete accumulation of disease (i.e., tumor spheroids) or single chemotherapy-resistant ovarian cancer cells floating in the ascites of OSPC-ARK-1 xenografted animals, to be specifically targeted by the FITC-conjugated CPE peptide (Figure 5).

**Table 2:** Biodistribution of FITC-CPE peptide in SCID mice with OSPC-ARK-1 tumor xenografts

Tumor-to-Organ Ratios	12 hours	24 hours	48 hours
Spleen	0.00	0.03	0.02

Liver	0.00	0.02	0.03

Kidney	0.07	0.10	0.08

Lung	0.06	0.05	0.11

Brain	0.00	0.15	0.01

Heart	0.01	0.01	0.00

**Note**: Mice were injected with 100 μg of FITC-CPE peptide i.v. and fluorescence in tumor and normal tissues was measured using a whole-body optical imaging system at the indicated time points as described in the Methods. Data are presented as tumor-to-organ ratios calculated from the organ distribution of FITC-CPE peptide in SCID mice with OSPC-ARK-1 tumor xenografts (n = 3-5 animals). The autofluorescence background in mice that did not receive fluorescent compound was subtracted from the experimental values. Results are representative of 2 independent experiments showing similar results.

## Discussion

Claudin-3 and claudin-4 are tight junction proteins overexpressed in ovarian carcinomas [3-13], the most lethal gynecologic malignancy in the United States [1,2]. Because claudin-3 and claudin-4 have been recognized as the receptors for *Clostridium perfringens enterotoxin *(CPE), these findings imply that ovarian cancer refractory to standard treatment modalities may be susceptible to CPE-based therapeutic approaches. Consistent with this view, in the last few years CPE structure/function relationship has been extensively investigated, mainly by characterizing the functional properties of enterotoxin fragments and point mutants [14-16]. In this regard, it has been demonstrated that the CPE_290-319 _COOH-terminus fragment is sufficient for high affinity binding to target cell receptor and small complex formation, although this fragment is incapable of initiating large complex formation and cytolysis [14-16].

In this study, we have taken advantage of the functional properties of enterotoxin fragments and we have first confirmed at both RNA and protein levels a high expression of the CPE receptors in multiple primary ovarian carcinomas characterized by high *in vitro *and *in vivo *resistance to chemotherapy. Secondly, using these freshly established biologically aggressive ovarian tumor cell lines, we have carefully assessed the accuracy and specificity of the binding of the carboxy-terminal fragment of CPE conjugated to a fluorescent tag (i.e., FITC-CPE peptide) as well as its internalization properties *in vitro *as well as in *in vivo *experiments using a highly relevant clinical model of chemotherapy resistant freshly explanted human ovarian cancer in SCID mouse xenografts.

Our results established, to our knowledge for the first time, that the CPE peptide is capable of specifically targeting chemotherapy resistant ovarian carcinoma cells overexpressing the CPE receptors claudin-3 and claudin-4. However, it did not bind to normal tissue cells or tumors not expressing significant levels of claudin-3 and/or claudin-4. These findings clearly indicate that the CPE peptide possesses specific targeting and/or homing properties for chemotherapy-resistant ovarian tumors. The remarkable tumor-homing efficiency of the peptide may be due to its propensity to be internalized by cells. Indeed, cells that bind CPE peptide due to the overexpression of the claudin-3 and claudin-4 receptors may transport it across the cell membrane and into the cytoplasm. Thus, peptide internalization is likely to contribute to its effectiveness in becoming concentrated in the targeted tumors. In this regard, it is worth noting that the CPE peptide used in our experiments was conjugated to fluorescin, a complex organic molecule with 44% of the molecular mass of paclitaxel [21]. While we found the fluorescence intensity to be time- and dose-dependent, all tumor cell lines tested exhibited internal FITC, thus demonstrating efficient accumulation of the fluorescent compound. These results, obtained in fresh unfixed tumor cells exclude the possibility of the peptide being artificially internalized during fixing. Furthermore, none of the cell lines exhibit significant auto-fluorescence. Of interest, under high magnification, a punctate fuorescent pattern was observed, which suggest, similarly to the data we have recently reported using an anti human claudin-3 scFv [19], that the CPE peptide may be compartmentalized after entry into cells. Interestingly, this punctuate pattern is similar to that previously observed with internalized epidermal growth factor, which enters via receptor-mediated endocytosis [22].

Occult micro-metastatic chemotherapy-resistant ovarian disease, by definition, is not detected by even the most sophisticated imaging techniques such as standard MRI and positron emission tomography (PET). To determine whether CPE peptide localizes to tumor, SCID mice harboring established peritoneal carcinomatosis of OSPC-ARK-1-derived tumor xenografts were injected i.v. with FITC-conjugated CPE peptide. Importantly, tumor explanted 12, 24 and 48 hrs after peptide injection showed fluorescing tumor cells while untreated tumor cells did not significantly autofluoresce. Moreover, brain, heart, lungs, kidney, spleen and liver from FITC-conjugated CPE peptide injected tumor bearing mice showed little labeling. A similar result was also obtained with FITC-conjugated CPE peptide injected tumor-free mice, which suggested that the inefficient labeling was not attributable to peptide depletion by the tumor. Remarkably, in our *in vivo *experiments, we found even discrete accumulation (i.e., tumor spheroids) or single chemotherapy-resistant ovarian cancer cells floating in the ascites of OSPC-ARK-1 xenografted animals to be specifically targeted by the FITC-conjugated-C-CPE peptide. These experiments suggest that CPE peptide may have a remarkable ability to target very early stage of occult metastasis foci and/or recurrence disease by detecting concealed tumor cells before they become clinically evident *in vivo*.

Eradication of occult micrometastases of chemotherapy-resistant disease by coupling radioactive isotopes or anticancer agents to the CPE peptide would be greatly beneficial in protecting patients with microscopic residual disease or small-volume macroscopic cancer resistant to standard chemotherapeutic agents. Consistent with this view, the potential of the CPE peptide as a carrier to specifically deliver therapeutic drugs *in vivo *in patients harboring incurable chemotherapy-resistant ovarian disease or other human malignancies overxpressing the CPE receptors such as breast, prostate, lung, endometrial, thyroid and pancreatic cancer [20,23-25] is further strengthened by the fact that it is nontoxic (no histological evidence of organotoxicity was observed in *in vitro *experiments or in CPE-peptide injected mice), while is able to behave as a shuttle during transit, and accumulates efficiently within the tumor in a few hours. As a result of our experiments, efforts to conjugate the CPE peptide to diagnostic and therapeutic radionuclides are currently in progress in our laboratory.

## Conclusions

Our results demonstrate for the first time that the CPE peptide binds and internalizes in ovarian tumor cells and therefore possesses excellent targeting or homing properties for the early diagnosis and/or treatment of chemotherapy-resistant ovarian carcinoma. These data combined with the high expression of claudin-3 and -4 receptors recently reported in other biologically aggressive epithelial human tumors, such breast, prostate, lung, endometrial, thyroid and pancreatic cancer [20,23-25] provide further evidence to suggest that CPE peptide may have great potential for diagnostic and/or radiometabolic therapy of ovarian carcinomas as well as other aggressive human tumors after labeling with suitable chemotherapeutics, radionuclides or toxins.

## Abbreviations

OSPC: ovarian serous-papillary carcinoma; CPE: Clostridium Perfringens Enterotoxin; FBS: fetal bovine serum; mAb: monoclonal antibody; scFV: single chain fragment variable; PBL: peripheral blood lymphocytes; q-rtPCR: quantitative real-time polymerase chain reaction; FITC: Fluorescein isothiocyanate.

## Competing interests

The authors declare that they have no competing interests.

## Authors' contributions

EC, FC, SB, MB, PT carried out the molecular in vitro studies including RT-PCR, flow cytometry and cell binding assays. EC, CR, JH, HF, MM, GM carried out the in vivo molecular studies with the CPE peptide in mice harboring multiple ovarian cancer xenografts. PS, DS MA, TR, MA-K, SP and AS participated in the design of the study and drafted the manuscript. AS conceived the study. All authors read and approved the final manuscript."

## Pre-publication history

The pre-publication history for this paper can be accessed here:

http://www.biomedcentral.com/1471-2407/10/349/prepub
